# Sounding the Alarm: Health in the Anthropocene

**DOI:** 10.3390/ijerph13070665

**Published:** 2016-06-30

**Authors:** Colin D. Butler

**Affiliations:** 1 Faculty of Health and Health Research Institute, University of Canberra, Canberra 2617, Australia; colin.butler@canberra.edu.au; Tel.: +61-2-6201-2194; 2 National Centre for Epidemiology and Population Health, Australian National University, Canberra 0200, Australia

**Keywords:** Anthropocene, limits to growth, planetary boundaries, health inequalities, eco-social tipping points, future health, Health-Earth

## Abstract

There is growing scientific and public recognition that human actions, directly and indirectly, have profoundly changed the Earth system, in a still accelerating process, increasingly called the “Anthropocene”. Planetary transformation, including of the atmosphere, climate, ecosystems and biodiversity, has enormous implications for human health, many of which are deeply disturbing, especially in low-income settings. A few health consequences of the Anthropocene have been partially recognized, including within environmental epidemiology, but their long-term consequences remain poorly understood and greatly under-rated. For example Syria could be a “sentinel” population, giving a glimpse to a much wider dystopian future. Health-Earth is a research network, co-founded in 2014, which seeks, with other groups, to catalyse a powerful curative response by the wider health community. This paper builds on a symposium presented by Health-Earth members at the 2015 conference of the International Society for Environmental Epidemiology. It reviews and synthesizes parts of the large literature relevant to the interaction between the changing Earth system and human health. It concludes that this topic should be prominent within future environmental epidemiology and public health. Created by our species, these challenges may be soluble, but solutions require far more understanding and resources than are currently being made available.

## 1. Introduction

It has become almost commonplace to hear that we live on a human-dominated planet, marked by immense environmental and social changes. The composition of the atmosphere has been altered at the global scale, in turn changing the climate. As the oceans warm and the glaciers melt, the sea level rises at an ever increasing rate. This is despite an increasing capture of water on land, itself mostly intentional and for human use [1]. A key driver of these planetary changes has been population growth, made possible by ingenuity and the rapid spread of innovation. The harnessing of extra-somatic energy, initially from the burning of biomass and the use of draft animals, and more recently from fossil fuel burning are additional drivers of population growth related planetary change.

Human population has increased from about 200 million 2000 years ago [2] to almost 7.5 billion today [3], a multiple of almost 40. It has doubled since about 1970, when its rate of annual increase peaked at over 2%. Since then the growth rate has fallen, but world population still rises by over 80 million per annum [3]. The increase in human population (and of energy use and invention) has also been made possible by transformation of the biosphere to meet human wants and needs. The biosphere refers to the thin film beneath, on and above the surface of the Earth (including soil, oceans, and atmosphere) in which interaction occurs between living species and non-living elements [4,5]. Much of this transformation has been deliberate. Vast forests have been exchanged for plantations and paddy fields. Much of the American prairies, for example, have been ploughed, though parts of the Russian steppe have been restored [6]. This suggests not only that adverse environmental change can be reversed in some settings but also that human values and attitudes may evolve in a more “biosensitive” way [7].

Some planetary and Earth land surface transformations have been indirect, by the introduction of new species [8] such as rabbits and foxes in Australia. Another example are “novel entities” such as endocrine disruptors and chlorofluorocarbons. Originally thought of as benign, chlorofluorocarbons significantly damaged the stratospheric ozone layer, on a planetary scale [9,10].

Smil has reviewed data suggesting that the combined biomass of humans and the animals that humans have domesticated outnumbers that of wild mammals by at least 35:1 [2]. The same review estimates that there are fewer than 50,000 bison left in the US, and less than half a million African elephants [2]. The numbers and composition of marine species have also been significantly altered, with large fish disproportionately harvested, at least for some species and in some regions [11]. In some marine ecosystems the numbers of “pest” species such as jellyfish, have reached plague proportions, leading to surprises including the temporary disablement, in 2006, of the recently commissioned aircraft carrier, the USS Ronald Reagan, when many jellyfish were sucked into the ship’s cooling water condensers [12].

As the numbers, security, and freedom of most large animal species on Earth has plummeted, human life has flourished, in an approximately equal exchange. Despite an uptick in conflicts such as Syria, human life expectancy (globally) has increased significantly, both absolutely and if adjusted for healthy years of life [3,13]. Most health experts, governments and leading organizations appear to accept that human progress will continue, despite ongoing decline in global environmental conditions [14].

For example the most recent revision of the median projection of global human population in 2100, issued by the United Nations Department of Economic and Social Affairs, is for over 11 billion people to be alive in 2100. Life expectancy at birth by then is forecast to have risen to 83 from its recent (2010–2015) level of 70 years [3]. This confidence, or perhaps complacency, is consistent with decades of earlier reports, particularly since about 1980 [15]. Until very recently, similarly optimistic views were expressed implicitly or explicitly by leading institutions such as the World Bank, the International Monetary Fund, the G8, and most corporations [16]. This confidence is understandable, given that few governments or organisations want to erode public confidence in the global system.

However, a minority of scientists and futurists have expressed deep concern, attempting to sound the alarm for the future of our species. The most obvious pathway to the impoverishment or even the annihilation of humans is via widespread conflict involving nuclear weapons [17]. Slower pathways to bleak futures, ending in impoverishment, lower numbers, and lower living standards are plausible, such as regional collapses in governance and public health [18]. Cutaneous leishmaniasis, for example, has become more common in Syria following the start of its civil war [19].

Steven Pinker has argued, persuasively, that violence by humans against humans (though not nature) has fallen sharply in recent decades [20,21]. However, his explanation for this as the rise of “reason” and his expectation that these trends will continue is less convincing. With so many natural resources in decline, and with population still rising it seems very unlikely that co-operation will continue to expand on the scale needed. There are now dozens of counterexamples, from the Central African Republic to the South China Sea.

One of the most authoritative warnings of potential global collapse is by Martin Rees, for five years President of the Royal Society. His book about this is starkly entitled Our Final Century (or in the U.S. edition “Our Final Hour”) [22]. There are many other cautions from significant figures, including Mark Carney, Governor of the Bank of England [23] and Pope Francis [24]. Admonitions have also come from a few with a health background [25,26,27], including Tony McMichael, a former president of the International Society for Environmental Epidemiology [25,28].

More broadly among the health community, such counsel has had little effect, with exceptions such as some conferences, editorials, and the Wellcome Trust’s “Our Planet Our Health” grant round, announced in 2015. Although most health curriculi includes environmental health very little such training concerns *systemic* environmental (and “eco-social”) risks, including the relationship of these adverse global environmental changes and factors with conflict. Even within political science, few analysts accept that the civil war that started in Syria in 2011 has significant environmental as well as social roots, such as the intensifying drought and groundwater shortage [29,30,31]. High human population growth also arguably had a role in Syria, by accelerating local resource depletion including of groundwater and by reducing the “demographic dividend” which, some analysts have long argued, helps to promote economic and human development [32,33], including through improved education [34].

Kelley et al. (and others, including in the military) have argued that climate change intensified the Syrian drought [30]. Climate change can be conceptualized as a “risk multiplier” for conflict, not only in Syria, but in Darfur and Somalia [31,35], and in many unsettling future scenarios. Via this web of eco-social factors, Syria can be conceptualized as a “sentinel” population, a case study which is likely to be replicated in broad principle, though not in precise detail, if, as seems likely, sufficient future stresses occur.

Two recent papers and a commentary warn that life in parts of the Middle East and North Africa, especially near the Persian Gulf, will become increasingly difficult under some plausible climate change scenarios by 2100, due to the combination of rising temperatures and humidity [36,37,38]. Although air conditioned life inside may be possible, how can the infrastructure be maintained? Will robots be sufficiently advanced? Will workers tending infrastructure do so only at night?

One of these papers warns of the impact on Haj pilgrims, which (due to its timing being determined by a lunar calendar) periodically occurs during the hottest months of the year [36]. Abstaining from water during hot days, a requirement of Ramadan (also determined by a lunar calendar), could also become extremely problematic to health in hot and humid conditions.

One of these articles, led by the director of the Max Planck Institute for chemistry also anticipates:

“that climate change and increasing hot weather extremes in the Middle East and North Africa, a region subject to economic recession, political turbulence, and upheaval, may exacerbate humanitarian hardship and contribute to migration”.

[38]

### About This Paper

Most of these warnings about the future note deteriorating environmental foundations [25], and argue that these will increasingly affect our own species, via cascading social and political changes. The literature on these topics, outside health, is too large to systematically review here. While the health impacts from planetary transformation are an emerging paradigm [27], the main purpose of this paper is to distil (and introduce to some) key relevant concepts to readers whose predominant training is in health, and for whom this literature is unfamiliar.

The terms and concepts which this paper will discuss are the “Anthropocene”, the “Earth system”, “Planetary Boundaries”, “Limits to Growth”, and “eco-social tipping points”. The article will consider the health implications of these interacting phenomena, including for disadvantaged populations before presenting conclusions. It will also describe Health-Earth, a research and advocacy network formed in 2014, which seeks to advance knowledge and understanding of these issues within the health community.

## 2. The Anthropocene

In 2011 the Geological Society of London met to explore the evidence for, and meaning of, the “Anthropocene” [39]. This term, coined in the 1980s by the biologist Eugene Stoermer [39] first appeared in the literature in 2000, in an article in the *Global Change Newsletter* [40]. Its authors (Stoermer and Nobel Laureate Paul Crutzen) suggested that Anthropocene be used to succeed the Holocene (the 10–12,000 year period since the last Ice Age) as recognition that we are now in a new geological epoch. They argued that incorporation of “Anthro” into a geological term would recognise the Earth-changing power of humanity not only for global geology but also ecology.

This term (Anthropocene) is not yet widely accepted or recognized but it has attracted interest from many disciplines. Two journals use its name (Anthropocene and The Anthropocene Review). Its supporters hope that a future international Geological Congress will adopt the Anthropocene, as its predecessor in 1885 decided to use the term “Holocene” 52 years after that term was coined [40]. If so, the term will escape its current narrow circle of familiarity.

Such dissemination, were it to occur, could have other benefits. Some have hope for its educative and potentially transformative nature:

“Teaching students that we are living in the Anthropocene, the Age of Men, could be of great help. Rather than representing yet another sign of human hubris, this name change would stress the enormity of humanity’s responsibility as stewards of the Earth. It would highlight the immense power of our intellect and our creativity, and the opportunities they offer for shaping the future”.

(quoted by Revkin [39])

Others however, are more critical. The term’s attraction has been complicated by debate concerning the date of its origin. The originally proposed period was in the 18th century when concentrations of the main greenhouse gas, carbon dioxide (CO_2_) started to rise, mainly due to increased coal burning. But the start of the Anthropocene has since been proposed to vary from when hominims commenced using fire [41], to as recently as 1945, when the first nuclear weapon was exploded [5]. It also been argued that the entire Holocene can be conceptualized as the Anthropocene [42].

The term has also been criticized for conflating the actions of a small minority of people (the most privileged and powerful) with the entire human species [43]. That may be plausible for some aspects of the Anthropocene, such as fossil fuel use and associated climate change, but is less convincing for other large scale global changes including species loss, deforestation, and the cumulative impact of countless smoky fires used for cooking, which, collectively, act to reduce the penetration of sunlight and to partly counter atmospheric warming.

## 3. The “Earth System”

Earth System science fits a *complex system* perspective, in which its linked elements are characterized by feedbacks, non-linear interactions, and thresholds (phase changes, or emergences). There are many examples of Earth system feedbacks, both reinforcing and stabilizing. Many examples of reinforcing feedbacks include in the Arctic, as it warms, as ice melts, and as trees spread north, in turn driving more warming, as vegetation and open water each absorb more heat than reflective ice. Arctic warming may also release currently stable stocks of carbon, stored in the tundra and in the ocean as methane hydrates [44].

Examples of phase changes are when water solidifies at freezing point or when we awaken. Social phase changes also occur, such as when an audience stands to applaud, or when a crowd in an uncovered stadium flees a violent storm. The onset of open hostility is another example of a social phase change. “Eco-social” changes can also occur, and are discussed below.

Examples of Earth system threshold points and phase changes include the switch from ice ages to interglacial periods. Earth system scientists have identified many potential future tipping points, including in the climate system. Examples include the transition to an ice-free Arctic [45], or the transformation of forests via droughts fires and logging [46,47]. Barnosky et al. (2012) have warned of a “state shift in Earth’s biosphere” [48].

Oldfield and Steffen argue that the scientific approach (most associated with the philosopher of science Popper) that involves the framing and testing of potentially refutable hypotheses that can be defined and tested is poorly suited to explore the challenges posed by an Earth System [49]. They also argue that many simple cause and effect relationships do not apply.

## 4. Planetary Boundaries

The term “planetary boundaries”, like the Anthropocene and the Earth system, also recognises and reflects the global scale of human alteration of the biosphere. This term first became prominent in 2009, when two linked papers outlined the concept of a “safe operating space” for humanity [50,51]. These ideas of safety are closely related to the concept of the Earth system and the rarely observed, though often called for precautionary principle (the community should wait for widespread consensus of safety before introducing new technologies and social practices) [52].

Planetary boundaries are not located at what are thought to be critical biophysical thresholds, but well up-stream, thus allowing a safety margin. However, the size of this safety margin is not known with certainty. Originally, nine Earth system processes, each of which was clearly being modified by human action, were included. These are shown in Table 1, in alphabetical order, together with their main identified health effects, both beneficial and harmful.

It is worth stressing that the processes which have led to the approach to planetary boundaries have been beneficial to humanity but have passed a “sweet spot” and have become problematic due to their immense scale. It is also important to mention some of these health problems exist at levels far short of the putative planetary boundary (itself buffered from the critical Earth system threshold, as mentioned).

Planetary boundaries do not identify health risks per se, but the argument of Earth system scientists is that if a sufficient number of boundaries are exceeded then profound effects for the Earth system will occur. If so, it follows that Earth’s social system (hence “eco-social”) and global health will be also profoundly harmed. The updated planetary boundaries report also identified two core boundaries (climate change and biosphere integrity) each of which is argued to have the potential on its own to force a new Earth System state should their boundaries be substantially and persistently transgressed.

Few if any of these processes exist in isolation. For example, land use change is related to biodiversity loss and to global warming; and CO_2_ accumulation drives ocean acidification. Atmospheric aerosol loading is related to climate change, and can act as both a stabilizing and reinforcing feedback. Atmospheric haze reduces sunlight penetration and heating [53], but black carbon particles (from incompletely combusted biomass or fossil fuels) can trap heat and accelerate glacier melting [53,54].

Table 1 identifies important but separated risks from the passing of local and planetary boundaries. Importantly, these are likely to be dwarfed by the combined *systemic* effects. McMichael grappled with ways to convey these consequences to skeptical health audiences, writing at one point

“We talk about ‘life support’ systems, but, frankly, the idea that the survival of *Homo sapiens* depends upon the sustaining of ecosystems still seems a bit far-fetched” [25].

Even now, more than two decades after his lament, few health workers appear to have grasped the immense significance of humans’ alteration of the planet, likely to unfold in the coming decades.

## 5. Limits to Growth

The term “limits to growth” was popularized by the best-selling book of that name, commissioned by the Club of Rome and published several years later in 1972 [70]. It took a systems approach and presented a computerized simulation of the interactions between Earth and human systems; it became one of the most controversial and discussed publications of the 1970s. The book warned that, without radical reform, civilization faced collapse, perhaps by the middle of the current century. This report was initially regarded as credible, but as the 20th century progressed, its influence waned. By the close of the 20th century, its central message was widely dismissed [71,72,73,74].

The abstract of an early article on planetary boundaries [51] claimed that planetary boundaries moved away from earlier work on limits to growth [70] which had an “essentially sectoral analysis”. However, in the body of that paper it is acknowledged that “our proposed framework builds on and extends approaches based on limits-to-growth”. In my view, this second formulation is more fair; the two concepts have considerable overlap, even though one is more contemporary [73].

While the original work on limits to growth had little discussion of climate change, it recognized that population growth, in combination with accelerating industrialization, in turn triggering growing resource scarcity, environmental decline had the capacity to lead to “overshoot and collapse” of civilization in a systems framework [70,74]. Climate change is not always thought of as a form of pollution, perhaps because the gases that largely drive it are invisible. However, it clearly is in substantial part a consequence of atmospheric contamination from the burning of fossil fuels such as coal and oil, aggravated by forest clearance and agriculture [64]. There are, of course, many other forms of pollution, local and regional, including novel entities ranging from endocrine disruptors [62] to radiation.

Another important aspect of limits to growth is the increasing scarcity of resources, including the scarcity of affordable energy for use by industry, individuals and households. This aspect has received some public health attention, including a special issue in the American Journal of Public Health [75]. In recent years, the price of oil has fallen (from a peak of over US $140/barrel in 2008 to approximately US $50 today), leading some commentators to argue that such concerns are overstated. However, few experts argue that the current comparatively low price of oil can be long sustained.

The main oil field in Saudi Arabia, Gharwar (still a critical source of supply), was reported in a 2014 paper as being injected with seven million barrels of sea water per day, a technique that maintains production but accelerates decline and may even reduce ultimate production [76]. Furthermore, even if additional oil reserves can be found, their exploitation should be greatly limited by the increasingly acknowledged imperative to slow the rate of CO_2_ accumulation [77].

This dilemma illustrates a less appreciated limit to growth. On the one hand, there may not be enough accessible, easily recoverable oil to fuel the global economy for more than a few more decades. But even if there is, the global capacity to safely absorb the carbon emissions released by the burning of this oil is itself critically limited. There is growing academic recognition, and also from some in business that much of the world’s oil resources are effectively unburnable [77,78], or they would be in a world that prioritises the avoidance of highly dangerous climate change and the acceleration of the clean energy transition.

Other forms of worsening resource scarcity include of phosphate, rare earths, helium and some metals. Additional aspects include a limit to the growth of crop yields [79], falling returns to increasing complexity [80], and imperfect co-operation. See Butler, 2015, for further review [81].

A clean energy transition (especially using solar and wind) might allow humanity to keep within its carbon budget (the amount of carbon which can still be burned if we are to avoid critically dangerous climate change) [78]. Geo-engineering, another form of ingenuity, has also been proposed as a way to contain the harm of climate change, even if the carbon budget is exceeded [82]. However, to ignore these issues is an imprudent violation of the precautionary principle. It could even be described as recklessly gambling with the fate of civilisation, and thus population health.

We may be in more danger than most appreciate. Climate change is accelerating, and a recent paper suggests the potential for rapid sea level rise is still underestimated [83]. In 2015, the average global level of CO_2_ exceeded 400 parts per million (ppm) and in late April 2016 it exceeded 404 ppm. (Its annual crest is in May). The increase in 2015 (2.9 ppm) was the highest ever [84]. There is growing concern that some of this increase is from nascent feedbacks, releasing stored carbon [44].

In the lead-up to the Conference of the Parties on climate change, held in Paris in 2015, the governor of the Bank of England, Mark Carney, warned, in a widely reported speech:

“The far-sighted amongst you are anticipating broader global impacts on property, migration, and political stability, as well as food and water security. … Past is not prologue … the catastrophic norms of the future can be seen in the tail risks of today”.

[23]

There are also limits to the human capacity to deal with multiple crises. Attempts to solve root causes can even be diverted by problems that arise from previous failures to tackle deep causes. For example, in March, 2016, it was reported that the European migrant and refugee crisis (mostly arising from the Syrian civil war) had temporarily displaced consideration of the Paris agreement on climate change from the agenda of a European Union meeting [85]. Although this was later disputed, it illustrates the finite capacity of policy makers to deal with multiple crises. Furthermore, the refugee crisis is itself arguably a consequence not only of climate change, but of other aspects of limits to growth (water scarcity, limits of social tolerance).

There are other examples. Global efforts to support the then newly announced Millennium Development Goals were diverted by the terrorist attacks on the U.S. in 2001, leading to the enormous cost of the wars in Iraq and Afghanistan. The G8 summit in Gleneagles, Scotland, which was intended to be focused on global poverty relief, was disrupted by the London terrorist attack which took place during the meeting [86]. Although the relationship between global poverty, inequality, and terrorism is intensely contested, they are also plausibly related [87], that is the 9/11 attacks in the U.S. may in some way have been contributed to by the extent of global inequality [88].

## 6. Eco-Social Tipping Points

The final concept to be introduced is “eco-social tipping points”. There is an increasing literature about environmental (or ecological) tipping points [45] and it is intuitively obvious to some that sufficient environmental change will inevitably induce, or be accompanied by, large-scale social change. However, despite this intuitive attraction, there is comparatively little recent scientific literature on this subject, particularly in association with the Anthropocene, Earth system science, Planetary Boundaries, and Limits to Growth, though it is implicit. For example, the subtitle of the Planetary Boundaries articles is a “safe operating space” not for the Earth system, but for “humanity” [50,51]. If the environment changes sufficiently, can society remain largely unaffected?

This reticence appears in part be a reaction against “environmental determinism”, the largely shunned idea, first formulated in the 18th century, that many social and historical phenomena are influenced (even “controlled”) by environmental factors and changes [89]. It is thus important to stress that social changes in response to major environmental shifts are not inevitable, but may occur. The concept is of importance if Limits to Growth and Planetary Boundaries are valid, because it means, beyond thresholds, such changes increase the risk to society, to health and, especially, to those who are poor and vulnerable. This will be explained in the next section.

Extreme interpretations in the other direction (i.e., that environmental determinants are *irrelevant* to human well-being) not only violate the precautionary principle but appear ideological. But, of course, relationships are not simple, nor always inevitable. Eco-social tipping points may exist, but they can be avoided.

## 7. The Disproportionate Risks of Adverse Global Environmental Change to the Poor

There is increasing recognition that inequality is increasing, within many countries. The phrase “the 1%” has become popular, reflecting the rise of inequality in the US and many other developed countries [90]. In contrast, the trend of global inequality in recent decades is disputed [87,91,92,93]. However, even optimistic analysts agree that the current global Gini co-efficient (even where income is increased by adjustment for purchasing power) exceeds 0.6 [93]. This level of inequality exceeds that of any single nation, and is far higher than most [87]. Furthermore, the level of global income inequality has been at least this unequal for decades, suggesting that the global poor survive in a trap of relative powerlessness [94].

Whatever the truth about the trend of global inequality, the material, nutritional [95], and health circumstances of the world’s poorest quintile is reported to have improved in recent decades. This is highly desirable, and will help prepare the poor for the risks to their health and well-being that the Anthropocene appears likely to deliver first to them, and perhaps then to us all. But it does not mean their indefinite health and security. It is the poorest who have contributed the least to the accumulation of greenhouse gases, who are most vulnerable to the health risks in the Anthropocene, via many pathways (see Table 2).

To argue that those who are wealthy and powerful have disproportionate influence over policy [87] both at national and international levels can also be criticized as ideological; nonetheless, from the perspective of the poor, the evidence is compelling. The G8 has never been led, nor is likely to be led, by countries such as South Sudan, Malawi, or Sierra Leone. The comparative lack of purchasing capacity and of international political influence of poor populations [87] is likely to persist, and will be accompanied by continuing vulnerability, including to the risks from encroaching limits, whether termed boundaries or another synonym. Indeed, the disproportionate risk of the poor to what McMichael called “Planetary Overload” [25] was identified by the current author as a cause of “environmental brinkmanship”, inspired by the term “nuclear brinkmanship” [87]. These ideas have recently been popularized by Naomi Klein, who explicitly links capitalism and inequality to the climate crisis [104].

Unfortunately, conventional proposals to lift the position of the poor generally involve more economic growth, as measured non-ecologically [105], with little recognition that, without extensive decoupling (i.e., loss of the relationship between environmental harm and economic growth) economic growth puts civilization closer to harmful thresholds. Conventional solutions to poverty paradoxically increase the risk to the poor; they must be leavened by vigorous attempts to reduce pressure on planetary boundaries and other limits.

The misery of most of the population in Syria can be conceptualized as a “sentinel”, a case study that may provide a glimpse into a dystopian future in which drought, aquifer depletion, and intractable local differences drive conflict. Although Germany has recently led Europe in giving hope to over a million refugees, many from Syria, who are being resettled its generosity is proving to be bounded. Europe is now following the example set by Australia in impeding routes to resettlement, even for people who have a right in international law to seek and be granted asylum.

## 8. Health-Earth and Other Solutions

Proponents of the recognition of planetary boundaries argue for use of the precautionary principle, suggesting that societies would be “unwise to drive the Earth System substantially away from a Holocene-like condition”, as this could lead, “with an uncomfortably high probability”, to a different state of the Earth System, that *is likely to be much less hospitable to the development of human societies* (emphasis added) [14]. These issues, even if venturing into fields of science most health workers are currently unfamiliar with, should be at the core of population health, as they are of such potential significance. If health scientists feel they lack the training to fully understand the arguments that lead to such conclusions, then they certainly lack the knowledge to dismiss them.

Although the Planetary Health Commission (supported by the Rockefeller Foundation and *The Lancet*) [27] is trying to explore the significance of this related work, much more is needed. For this reason, a research network called Health-Earth was co-founded in 2014. It seeks, with other actors, including from within civil society and the Earth system science community (including Future Earth and Future Earth Health) to catalyse a powerful response by the wider health community, to the issues raised in this paper.

Health-Earth was co-founded by eight individuals associated with interdisciplinary research groups from six countries and one UN University (see Table 3). Three co-founders (Butler, Ebi and Morse) were previously associated with the health dimension of the Earth System Science partnership, one of the predecessors of Future Earth. At the time Health-Earth was founded Future Earth lacked a health dimension.

A symposium was presented by three Health-Earth co-founders (assisted by Colin Soskolne) at the 2015 conference of the 27th International Society for Environmental Epidemiology. At the symposium, presenters argued that these topics should not only be prominent within future environmental epidemiology and public health, but within medical and health curricula. In 2016, the two founding co-chairs of Health-Earth were prominent in a seminar at the Finnish Parliament, called Arctic Environment, People and Health [106]. Created by our species, these challenges may be soluble, but solutions require far more understanding and resources than are currently being made available.

## 9. Conclusions

Understanding these linked issues is vital for enduring global population health. The topic is deeply unsettling. It is important that health workers who grapple with the implications of these topics do not transmit pessimism but, equally, they should not project complacency. A “social vaccine” could help, conveying sufficient concern (a social antigen) to motivate action, but not so much as to cause despair [107].

Many elements exist that give hope, including the rapid growth of renewable energy technology, which will soon slow the rate of climate change, provided unstoppable reinforcing feedbacks are not initiated. Accelerating the demographic transition, through human rights based means such as more education, will reduce poverty and health traps and will enable societies to better prepare for health in the Anthropocene [33]. Greater awareness of these issues by the health community should also contribute to the demand for effective solutions, but information is not enough. Even though well-off and powerful populations are comparatively insulated from the initial impacts of approaching planetary boundaries, their exceedance could drive down living standards and health for everyone. For this reason, the world needs urgent action, not just to protect environmental properties, but to protect human health, which, ultimately, is dependent on these foundations.

## Figures and Tables

**Table 1:** The nine original planetary boundaries and some of their health effects.

Planetary Boundary	Health Benefit	Main Identified Existing Health Harm
Atmospheric aerosol loading	Heating living areas, cooking food, electricity production	Respiratory and cardiac disease; [55] coal burning is a major source of mercury accumulation in the marine foodweb [56].
Biogeochemical flows (nitrogen and phosphate)	Increased soil fertility and food production, reduced pressure on forests (due to more intensive farming)	Can contribute to algal blooms reducing fresh water quality and quantity [57]. Coastal blooms may reduce fishery productivity, are unsightly and in some places (e.g., China) are expensive to remediate.
Loss of biological diversity	Human colonisation (e.g., in Arctic), [58] reduced predation, increased food production	Loss of potential pharmaceuticals and other valuable products, [59] potential “reprogramming” of some ecosystems towards more human and animal disease, such as transmitted by insect and mammalian vectors such as mosquitoes and bats [60,61].
Chemical pollution	Enables many modern technologies and materials	Endocrine disruption, cancer, birth defects, neurological conditions (including to sensitive sub-groups) [62]. Mining disasters including from failed tailings dams, Minimata disease, Bhopal, etc. [63].
Climate change	Industrialization enabled by climate change generating processes	Heat stress, disasters, some infectious diseases, regional food scarcity, population dislocation, conflict, mental health effects, and distress [64,65].
Freshwater use	Agricultural and industrial production, electricity production from dammed water	Scarcity can exacerbate “water washed” diseases (e.g., scabies, trachoma) [66] and also be associated with reduced water quality and thus diarrheal diseases; drought harms vulnerable farmers including their mental health [67].
Land system change	Increased food production	Reduced ecological integrity and climate change (see above), loss of livelihood and well-being for displaced populations [68].
Ocean acidification	See climate change	Potential to reduce quantity and quality of marine food, an important source of protein and micronutrients to humans [69].
Stratospheric ozone depletion	Firefighting chemicals and some fertilizers	Cataracts, skin cancer, both human and animal, including among domesticated animals [10].

**Table 2:** In numerous ways, financially poor populations are vulnerable to adverse consequences of adverse environmental change. They are thus vulnerable to many adverse health effects, including impaired mental health, suicide, and other forms of self-harm, injuries, renal impairment, multifactorial poor health, and infectious diseases. The named groups and mechanisms are a small subset of the total number.

Population	Vulnerability	Health Effect
Poor coastal, delta and island populations	Flooding, trauma, forced migration, financial exploitation [68]	Poverty, infectious diseases, post-traumatic stress disorder, multifactorial poor health
Construction workers in the Persian Gulf [36,38,94]	Heat stress, labor and financial exploitation [96]	Dehydration, renal impairment, accidents, sudden death [97]
Members of religious and ethnic minorities, e.g., Buddhists in Bangladesh, Muslims in Myanmar [96]	Violence, exploitation, forced migration [98]	Post-traumatic stress disorder, physical trauma and undernutrition
Haj pilgrims [36]	Heat stress [36,37]	Dehydration, renal impairment, accidents, infectious diseases such as Middle East Respiratory Syndrome [99]
Sub-Saharan Africans [35,38]	Forced migration	Drowning, abuse, trafficking, exploitation, infectious diseases, suicide [100,101]
Existing refugees	Discrimination, imprisonment, undernutrition, denial of health services	Injuries, multifactorial poor health, including of mental well-being [102,103]

**Table 3:** In 2014, eight individuals associated with interdisciplinary research groups, all with strong links to health, established the network Health-Earth (H-Earth) from six countries and one UN University eight co-founders of Health-Earth. Prof Colin Soskolne, not a co-founder, is an affiliate at the University of Canberra, Australia.

Name	Country	Chief Expertise
Al Delaimy, Wael	US	Environmental epidemiology, ethics
Butler, Colin	Australia	Public health, global change and health
Capon, Tony	UN system	Public health, global health
Ebi, Kristie	US	Epidemiology, climate change and health
Hancock, Trevor	Canada	Public health, urban systems
Jaakkola, Jouni	Finland	Environmental epidemiology, ethics
Morse, Andy	UK	Climate impacts, infectious diseases
Potter, John	New Zealand	Epidemiology
